# Quinia in Antiseptic Treatment

**Published:** 1881-05

**Authors:** Hugo Erichsen

**Affiliations:** Detroit, Michigan


ARTICLE IV.

### QUINIA IN ANTISEPTIC TREATMENT.

BY HUGO ERICHSEN, DETROIT, MICHIGAN.

The antiseptic treatment with carbolic acid has not always been successful. Billroth states, in his admirable surgical pathology, that "Lister's dressing often causes more or less severe poisoning by the annoying dermatitis induced by the carbolic acid." Sloughing has also often occurred in wounds treated with acidum carbolicum. Without going into details as to the action of carbolic acid, salicylic acid, etc., I will try to show the great value of antiseptic treatment by quinia. We find in Bartholow's valuable treatise on Materia Medica and Therapeutics the following:

"Cinchona is antiseptic; it arrests putrefactive decomposition and promotes healthy cicatrization. Quinia is very destructive of the minute organisms and will prevent decomposition."

M. Briquet informs us in his "*Traite Therapeutic du Quinanina et de ces-preparations*" that sulphate of quinia is not a stimulant, but a cooling and calming remedy.

According to Bing and Liebermeister, of Germany, quinia has a *specific* (see Stille, *Therapeutics and Materia Medica*) influence in checking the vital movements of the white corpuscles. It hinders their immigration through the vascular walls and checks the generation of new white corpuscles. Quinia reduces all the visible factors of suppurative inflammation.

Quinia sulphatis, dissolved in slightly acidulated water, or cinchona sulphatis, dissolved in boiling water may be used in the form of a spray. The wound is to be dressed as in Lister's method, except the substitution of carbolic acid by quinia sulphatis. The following benefits will be derived from the use of quinia in antiseptic treatment.

1. Decomposition going on in a wound will cause what is termed surgical fever; quinia, by preventing this decomposition, also prevents the fever.

2. Quinia not leaving the objectionable smell of carbolic acid will not produce vomiting or nausea.

3. Quinia promotes healthy cicatrization.

4. Quinia will destroy the minute organisms.

5. It will leave a sedative and cooling influence upon the wound.

6. It will prevent suppuration.

I beg the profession to employ quinia in antiseptic treatment and to report the result to the INDEPENDENT PRACTITIONER.

